# On the evolving nature of c/a ratio in a hexagonal close-packed epsilon martensite phase in transformative high entropy alloys

**DOI:** 10.1038/s41598-019-49904-5

**Published:** 2019-09-12

**Authors:** Subhasis Sinha, Saurabh S. Nene, Michael Frank, Kaimiao Liu, Priyanka Agrawal, Rajiv S. Mishra

**Affiliations:** Center for Friction Stir Processing, Department of Materials Science and Engineering, University of North Texas, Denton, TX 76207 USA

**Keywords:** Mechanical properties, Metals and alloys

## Abstract

Activation of different slip systems in hexagonal close packed (h.c.p.) metals depends primarily on the c/a ratio, which is an intrinsic property that can be altered through alloying addition. In conventional h.c.p. alloys where there is no diffusion-less phase transformation and associated transformation volume change with deformation, the c/a ratio remains constant during deformation. In the present study, c/a ratio and transformation volume change of h.c.p. epsilon martensite phase in transformative high entropy alloys (HEAs) were quantified as functions of alloy chemistry, friction stir processing and tensile deformation. The study revealed that while intrinsic c/a is dependent on alloying elements, c/a of epsilon in transformative HEAs changes with processing and deformation. This is attributed to transformation volume change induced dependence of h.c.p. lattice parameters on microstructure and stress state. Lower than ideal c/a ratio promotes non-basal pyramidal 〈c + a〉 slip and deformation twinning in epsilon phase of transformative HEAs. Also, a unique twin-bridging mechanism was observed, which provided experimental evidence supporting existing theoretical predictions; i.e., geometrical factors combined with grain orientation, c/a ratio and plastic deformation can result in characteristic twin boundary inclination at 45–50°.

## Introduction

Since the advent of complex concentrated alloys (CCAs) or multi-principal element alloys (MPEAs), various high entropy alloys (HEAs) have been studied across the world^1–4^. Recently, transformation induced plasticity (TRIP) effects, earlier known for steels^5–7^, were successfully introduced in HEAs. As a result, the ε-martensitic transformation and the presence of the hexagonal close packed (h.c.p.) ε phase in the microstructure of transformative HEAs gained importance rapidly^8–11^.

Our recent work introduced an alloy design strategy that involved responsive phase evolution, thereby stabilizing either thermodynamically stable γ or unstable ε phase in the as-cast condition itself, and giving rise to “Microstructurally Flexible HEAs^12,13^”. This microstructural flexibility was realized by synergistic variation of alloy chemistry, processing conditions and annealing treatments^12–14^. Excellent tensile properties were achieved in transformative HEAs processed by friction stir processing (FSP), a technique that involves the synergistic application of strain, strain rate and temperature^15^. Superior strength-ductility and work hardening response were possible through γ – ε dual-phase strain partitioning, as reported for CS-HEA^12^ and Al-HEA^13^. Besides, the significance of martensitic transformation for shape memory effect is well-known^16–20^. Thus, transformative HEAs provide opportunities to investigate the difference in the response of this h.c.p. structured phase compared with well-known h.c.p. metals. Additionally, how the f.c.c. to h.c.p. transformation contributes to strain accommodation during deformation of the material needs investigation.

In line with this, the c/a ratio of the h.c.p. crystal structure is vital to deformation mechanisms in h.c.p. alloys. Particularly, the non-basal pyramidal 〈c + a〉 slip and deformation twinning modes, which are crucial for accommodating higher strain along c-axis and associated work hardening behavior depend on the c/a ratio. Conventional h.c.p. metals like Mg or Ti have an intrinsic c/a that depends on alloy composition and does not vary because of processing or deformation. Very recently, Bu *et al*.^21^ reported c/a ratio of the epsilon phase in a Fe_50_Mn_30_Co_10_Cr_10_ dual-phase HEA. The present study investigates the c/a of ε (h.c.p.) phase in five transformative HEAs as a function of alloy composition (in the as-cast condition). Also, the effect of subsequent friction stir processing and deformation on the c/a ratio of the ε (h.c.p.) phase is investigated for all the alloys. Therefore, this study aims to elucidate the ε-phase controlled deformation mechanisms in transformative Fe-Mn-Co-Cr-Si containing HEAs through quantification of the c/a ratio of the ε (h.c.p.) phase and transformation volume change in these alloys.

## Results and Discussion

### Evolution of c/a ratio and transformation volume

**Figure 1:**
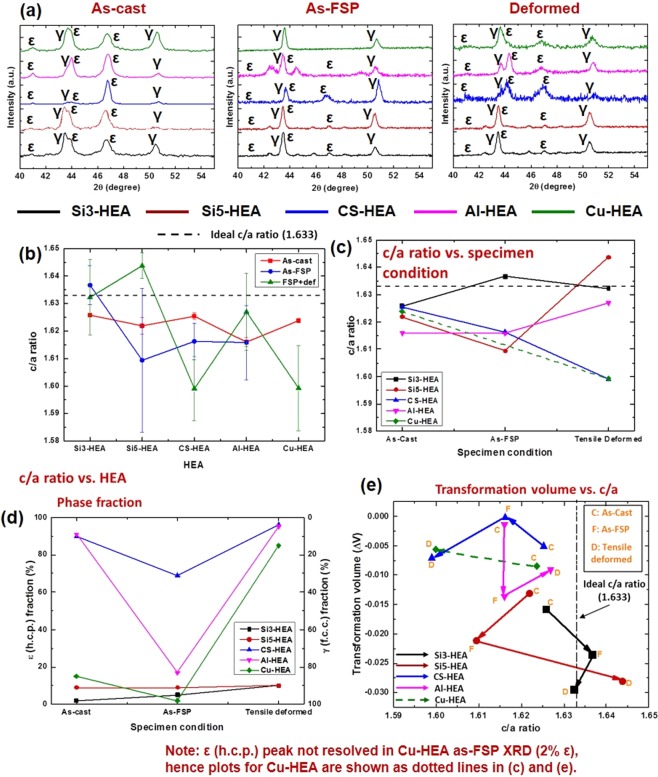
(**a**) XRD plots in various conditions; c/a ratio as a function of (**b**) HEA (**c**) specimen condition; (**d**) phase fraction as a function of specimen condition; (**e**) transformation volume as a function of c/a ratio.

**Figure 2:**
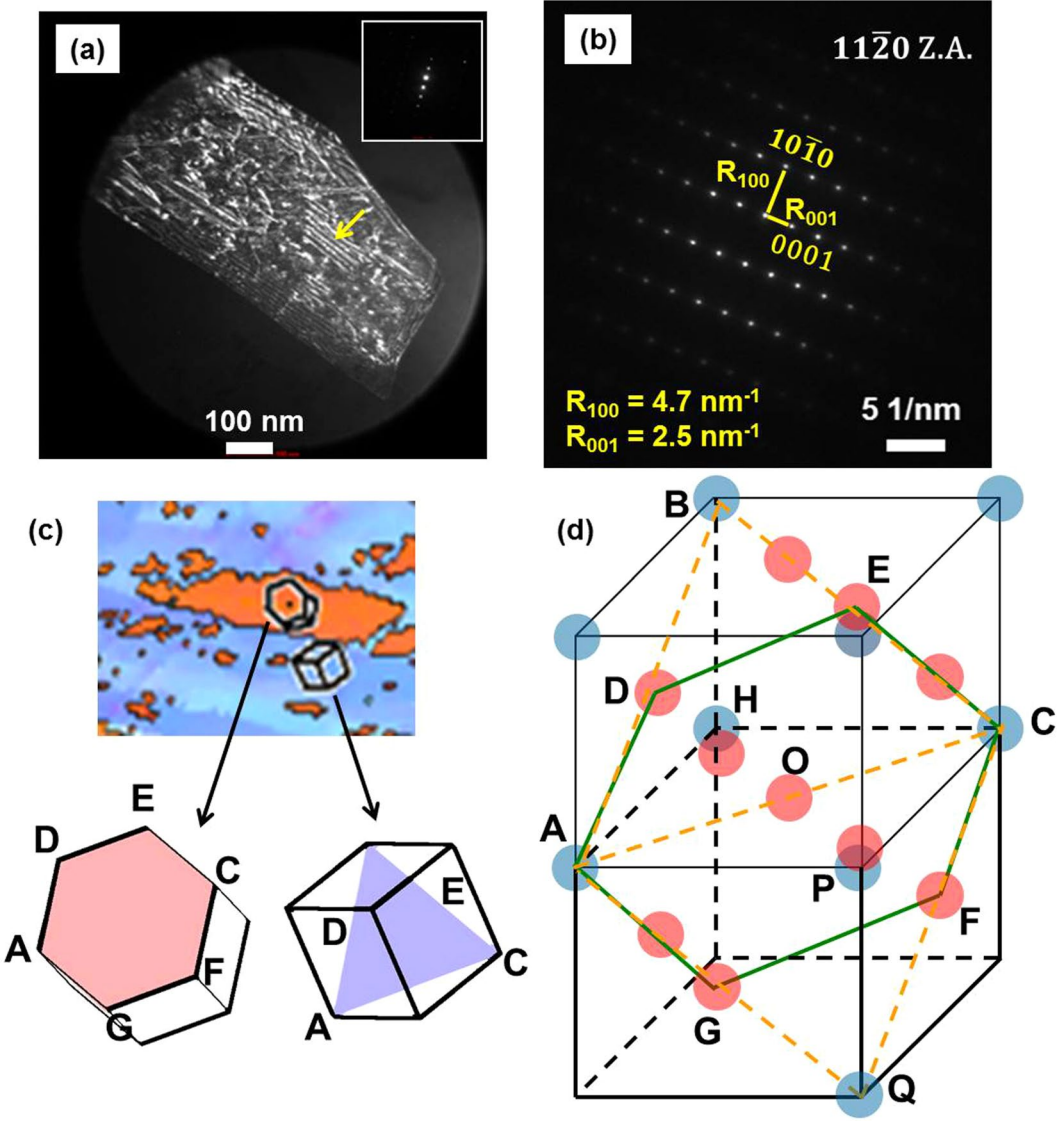
(**a**) Weak beam dark field TEM image to show stacking faults in as-FSP CS-HEA, (**b**) $11\bar{2}0$ zone axis TEM diffraction pattern from ε (h.c.p.) phase in as-cast CS-HEA for estimation of c/a ratio, (**c**) example of Shoji-Nishiyama orientation relationship (SN OR) from microstructure of as-cast Si5-HEA, and (**d**) schematic to explain SN OR.

The lattice parameters and c/a ratios of ε (h.c.p.) phase in five HEAs (described in Methods) in as-cast, as-FSP and tensile deformed conditions were obtained from the peak positions in X-ray diffraction (XRD) plots (Fig. 1(a)). Peak broadening was also analyzed from the XRD plots. The full width at half maximum (FWHM) for the ε (h.c.p.) peak at 2θ ≈ 47° increased from as-cast to as-FSP to deformed condition (‘ω’ values from Pseudo-Voigt function fitting are 0.39, 0.59 and 0.76 for as-cast, as-FSP and deformed CS-HEA, respectively). Similar peak broadening is also observed in Al-HEA and Cu-HEA from as-cast to deformed condition. This peak broadening is attributed to crystal lattice distortion (micro-strain) and stacking faults. Weak beam dark field imaging carried out on TEM foil obtained from as-FSP CS-HEA, confirms the presence of stacking faults as shown in Fig. 2(a) (marked by yellow arrow). Some peak shift can be also observed for γ (f.c.c.) peaks in Fig. 1(a), which could possibly be due to phase separation or chemical ordering. Figure 1(b) shows the variation of ε-phase c/a ratio in all five HEAs in various conditions. In the as-cast condition, the c/a ratio of the ε (h.c.p.) phase in these HEAs is similar to Mg. Bu *et al*. reported ε (h.c.p.) c/a = 1.616 in a Fe_50_Mn_30_Co_10_Cr_10_ dual-phase HEA^21^. Among the present HEAs in the as-cast condition, Al-HEA has c/a = 1.616, while others are slightly higher, although all have c/a less than ideal value of 1.633. Stanford and Dunne reported that in Fe-Mn-Si shape memory alloys, Si increases c/a ratio, while Mn decreases c/a ratio; however, the effect of Si is 25 times stronger than that of Mn^22^. Among the present HEAs, Si3-HEA has the lowest Si content and the highest Mn content. Yet, the c/a ratio of Si3-HEA is slightly higher than the other four HEAs. This difference from the shape memory alloys reported earlier^22^ is attributed to the presence of Co (which itself is h.c.p.) in these HEAs that influences the lattice distortion. The decreasing order of the c/a ratio is Si3-HEA > CS-HEA > Cu-HEA > Si5-HEA > Al-HEA. CS-HEA (c/a = 1.6254) is closest to Si3-HEA (c/a = 1.6258) due to the higher h.c.p. Co content. The c/a ratio of ε (h.c.p.) in as-cast CS-HEA was also verified from TEM diffraction pattern (DP) shown in Fig. 2(b) (c/a obtained from TEM DP was 1.628). Cu-HEA and Al-HEA have the same Co-content as CS-HEA, but the addition of f.c.c. stabilizers (i.e., Cu or Al) decreases c/a ratio. Among the latter two, Cu has smaller atomic radius and lattice constant than Al. Therefore, Cu-HEA shows only slightly reduced c/a than CS-HEA, while Al-HEA shows the lowest c/a in as-cast condition.

However, note that the difference in c/a ratio with alloy composition is least in as-cast condition and most in tensile deformed condition. Figure 1(c) shows that CS-HEA and Cu-HEA show decrease in c/a with processing and deformation, while Al-HEA shows increase in c/a. The trend in Si3-HEA is similar with CS-HEA, while the trend in Si5-HEA is similar with Al-HEA. The individual trends (of the different alloys) are attributed to the combined effect of alloy chemistry, responsive phase evolution, elastoplastic deformation characteristics and transformation volume change. Flexible microstructural evolution in these alloys with processing and deformation^12–14^ indicates that the change in c/a is related to microstructural evolution. Therefore, phase fractions as a function of specimen condition are shown in Fig. 1(d). Si3-HEA and Si5-HEA are γ (f.c.c.) dominant in all three conditions (as-cast, as-FSP and deformed). CS-HEA and Al-HEA exhibit most responsive phase evolution based on γ to ε transformation; while Cu-HEA transforms to ε-dominant microstructure after deformation although as-cast and as-FSP conditions have small fractions of ε-phase. Therefore, the latter three alloys are more interesting for studying ε-phase.

Comparing CS-HEA and Al-HEA in Fig. 1(d) confirms they have similar ε-fraction in as-cast and tensile-deformed conditions (both ε-dominant); however, in as-FSP condition both show dual phase microstructure, with higher γ in Al-HEA and higher ε in CS-HEA. Interestingly, c/a ratios of these two alloys are different in as-cast and deformed conditions, while the ratios converge to similar values in as-FSP condition (Fig. 1(c)). These observations indicate two important points. First, c/a ratio of ε (h.c.p.) is stabilized in dual-phase microstructure (with significant fraction of both phases) rather than microstructure tending towards single-phase. For the same reason, Si3-HEA and Si5-HEA also showed larger variation in c/a despite remaining γ-dominant with small change in ε-fraction as a result of FSP and deformation. Second, in the deformed condition, ε-fractions in CS-HEA and Al-HEA are almost similar but c/a decreases in CS-HEA, while it increases in Al-HEA. Therefore, the c/a ratio in deformed specimen also depends on whether the ε is just transformed ε, or deformed ε that has accommodated some strain. The as-FSP microstructure of Al-HEA had high γ-fraction (Fig. 1(d)). Relatively higher phase transformation occurred to reach 95% ε-fraction after tensile deformation^13^, but a significant amount of this ε was a newly-formed transformation product rather than as-processed ε. On the other hand, CS-HEA retained 69% ε-fraction after FSP, which then increased to 95% after tensile deformation^12^. Thus, lower phase transformation occurred and the microstructure was comprised of considerably work-hardened ε. Intrinsic c/a ratio is related to electronic configuration^23^. Therefore, conventional h.c.p. metals like Mg or Ti do not exhibit changes in their c/a ratio with annealing or deformation, except showing variation due to alloying^24^. Although c/a change with large strain was recently reported for tetragonal crystal structure^25^, c/a ratio is usually related to bond strength and elastic deformation. Therefore, c/a variations with deformation in the present study are partly related to elastoplastic deformation. CS-HEA and Al-HEA differ in the amounts of as-FSP ε and the amount of phase transformation taking place during deformation. The amount of ε undergoing elastoplastic deformation at low strain is different in the two alloys. Nanoindentation of as-cast CS-HEA and Al-HEA in our previous work^26^ illustrated that Al-HEA shows greater inherent resistance to deformation in the elastoplastic regime than CS-HEA (characterized by larger elastic regions between short displacement bursts in Al-HEA than CS-HEA). Therefore, higher volume of newly formed ε undergoes elastoplastic deformation in Al-HEA but the magnitude of distortion is low. Additionally, the effect of the volume change due to transformation must be taken into account.

The transformation volume change (ΔV) was calculated using Eqs (1–3)^22^.

1 $${{\rm{V}}}_{{\rm{fcc}}}={{\rm{a}}}_{{\rm{fcc}}}^{3},\,{\rm{where}}\,{{\rm{a}}}_{{\rm{fcc}}}={\rm{lattice}}\,{\rm{constant}}\,{\rm{of}}\,{\rm{f}}{\rm{.c}}{\rm{.c}}{\rm{.}}$$

2 $${{\rm{V}}}_{{\rm{hcp}}}=\frac{\sqrt{3}}{2}{{\rm{a}}}_{{\rm{hcp}}}^{2}{{\rm{c}}}_{{\rm{hcp}}},\,{\rm{where}}\,{{\rm{a}}}_{{\rm{hcp}}},\,{{\rm{c}}}_{{\rm{hcp}}}={\rm{lattice}}\,{\rm{constants}}\,{\rm{of}}\,{\rm{h}}{\rm{.c}}{\rm{.p}}{\rm{.}}$$

3 $$\Delta V=\frac{2{{\rm{V}}}_{{\rm{hcp}}}-{{\rm{V}}}_{{\rm{fcc}}}}{{{\rm{V}}}_{{\rm{fcc}}}}$$

The dimensional changes along c-axis and perpendicular to c-axis resulting from transformation induced volume change are important^27^. Additionally, there would be constraints imposed by the f.c.c.-h.c.p. crystallographic orientation relationship. Larger the volume change, greater would be the net decrease in dimension perpendicular to the c-axis to accommodate the volume change. Figure 1(e) shows ΔV plotted as a function of c/a ratio for the present HEAs in various conditions. Al-HEA shows increase in magnitude of ΔV from as-FSP to deformed state, while CS-HEA shows decrease in |ΔV|. Since CS-HEA shows lower |ΔV|, there is no change in a_hcp_ dimension from as-FSP to deformed state; while c_hcp_ decreases, resulting in lower c/a (Supplementary Table S1). Also note that, a_fcc_ does not change for CS-HEA from as-FSP to tensile deformed condition. On the other hand, Al-HEA with higher |ΔV| undergoes greater decrease in a_hcp_ and slight increase in c_hcp_, resulting in higher c/a., while a_fcc_ decreases (Table S1).

Earlier researchers reported volume expansion (positive ΔV) associated with f.c.c. to body centered cubic (b.c.c.) or body centered tetragonal (b.c.t.) martensitic transformation in ferrous alloys^28,29^; while, Stanford and Dunne obtained negative ΔV for f.c.c. to h.c.p. martensite transformation in six of their shape-memory alloys and one alloy showed “anomalous” positive ΔV^22^. Earlier work on 13% Mn steel also showed volume contraction for γ → ε transformation^30^. Recently, Wei *et al*. showed volumetric contraction associated with martensitic transformation in metastable Fe_45_Mn_35_Co_10_Cr_10_ HEA^31^. The present HEAs show negative ΔV, indicating volumetric contraction. However, the magnitude of ΔV represents the reversibility of the martensitic transformation and is important for shape memory effect^32^. And, while martensite reversibility was predicted from changes in c/a ratio previously^33^, transformation volume is a more appropriate measure of reversibility^22^. Lower |ΔV| corresponds to higher reversibility and shape memory effect. Based on |ΔV|, the present study shows that martensite reversibility is highest in CS-HEA. In general, CS-HEA, Al-HEA and Cu-HEA have lower |ΔV| compared to Si3-HEA and Si5-HEA, hence the former three HEAs are better candidates for shape memory effect.

The dimensional changes along a-axis and c-axis during f.c.c. to h.c.p. transformation are the origin of the stress-state dependence of c/a ratio. The Shoji-Nishiyama (SN) orientation relationship (OR) for γ (f.c.c.) to ε (h.c.p.) phase transformation is given by^30^

4 $${(111)}_{{\rm{\gamma }}}//{(0001)}_{{\rm{\varepsilon }}}\,{\rm{and}}\,{[10\bar{1}]}_{{\rm{\gamma }}}//{[11\bar{2}0]}_{{\rm{\varepsilon }}}$$

We also observed the SN relationship in the microstructure of as-cast Si5-HEA alloy in an earlier study^11^ (Fig. 2(c)). The γ (f.c.c.) to ε (h.c.p) transformation requires shear and shuffle of atoms^34,35^. The schematic in Fig. 2(d) shows two f.c.c. unit cells, one on top of the other. (111) plane of the top unit cell is marked with yellow dotted line (ABC). According to SN OR, when f.c.c. → h.c.p. transformation takes place, the (111) of f.c.c. becomes the basal (0001) of the h.c.p. and $[11\bar{2}0]$ direction of h.c.p. coincides with $[10\bar{1}]$ direction of f.c.c. Therefore, the possible orientation of the basal (0001) plane of h.c.p. is marked by green solid line (AGFCED). This comprises the face-center atoms D and E (red color) above the APCH plane and atoms G and F (red color) from the unit cell below, along with the corner atoms A and C (blue color).

As a result of this OR, the atomic arrangement of A, G, F, C, E and D would require angular distortion to attain the atomic arrangement of the h.c.p. basal plane but all these atoms are coplanar on the habit plane ((111) of parent f.c.c.). In contrast, the distortion required perpendicular to this plane to create the c-axis of the h.c.p. unit cell would involve displacement or shuffle of atoms that are out of the habit plane, in a direction perpendicular to the habit plane. Therefore, the latter distortion is more dependent on the stress-state. This anisotropic distortion makes c/a ratio sensitive to stress-state and ΔV corresponds with the relative dimensional changes along a-axis and c-axis. In short, f.c.c. to h.c.p. transformation involves ΔV that is dependent on stress-state and hence, c/a ratio of ε (h.c.p.) phase is responsive to processing and deformation.

### Contribution of slip modes to deformation

**Figure 3:**
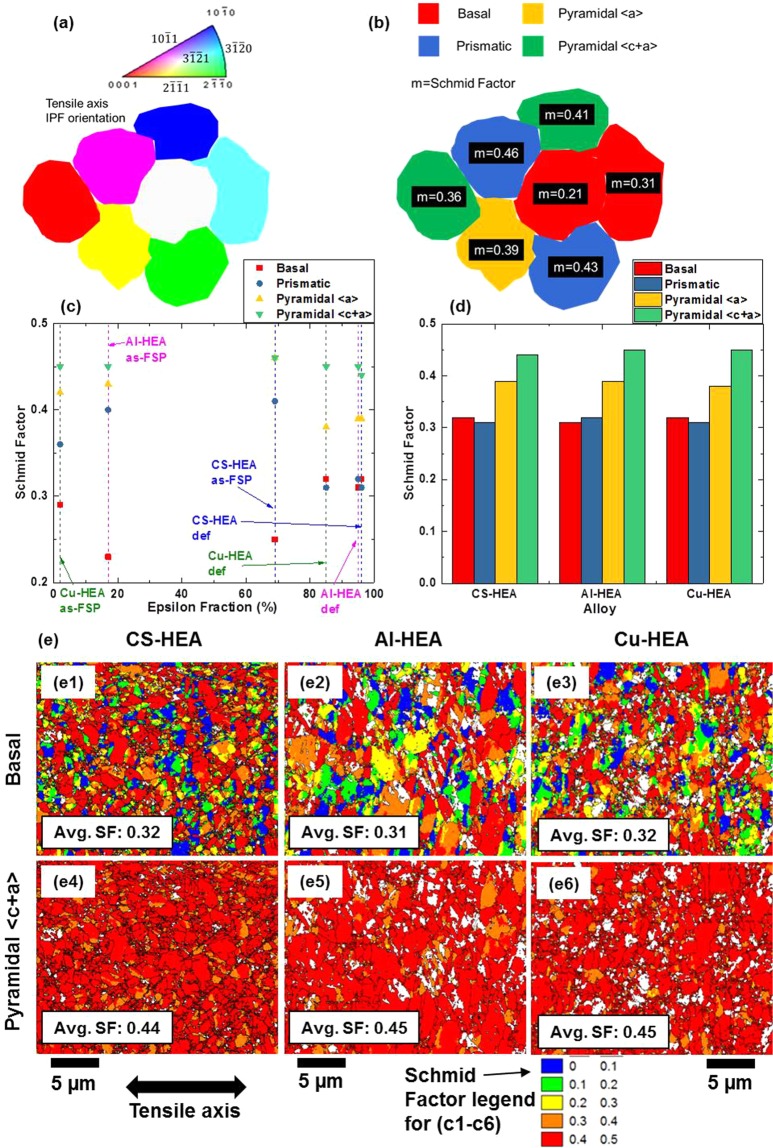
(**a**) A theoretical random distribution of grains colored by tensile axis IPF orientation, and (**b**) related Schmid factor distribution showing favorable slip system of theoretical grain orientation distribution in (**a**). Schmid factors (along tensile axis) of various h.c.p. slip modes in CS-HEA, Al-HEA and Cu-HEA presented as (**c**) function of ε fraction for comparison between as-FSP and deformed conditions, and (**d**) comparison between modes for each of the three alloys in deformed condition. (**e**) EBSD maps showing distribution of Schmid factor along tensile axis for basal and pyramidal 〈c + a〉 slip in deformed specimens of the three alloys.

Schmid factor (SF) indicates the propensity for activation of various deformation modes^36^, although non-Schmid twinning in h.c.p. metals is quite common^37^. SF analysis was performed for as-FSP and tensile-deformed specimens of CS-HEA, Al-HEA and Cu-HEA. Figure 3(a) shows a theoretical random distribution of grains colored by tensile axis inverse pole figure (IPF) orientation. For example, a grain with $[10\bar{1}1]$ along the tensile axis (TA) is colored magenta; another grain with [0001] along TA is colored red and so on. The favorable slip systems for the grains in Fig. 3(a) are shown in Fig. 3(b). The related SF distribution (Fig. 3(b)) is colored by the most favorable slip system (e.g. grains favoring basal slip are red) and the corresponding SF values are labeled on the respective grains. For instance, a basal oriented grain would have highest SF for pyramidal 〈c + a〉 slip, while $[10\bar{1}1]$ oriented grain would have highest SF for prismatic slip.

Figure 3(c) shows experimental SFs along tensile axis, plotted as a function of ε-fraction. The as-FSP specimens show larger variation in average SFs of the different slip modes compared to tensile deformed specimens. The smaller range of SF values in tensile deformed specimens (that have higher ε-fraction than as-FSP specimens) indicates a tendency to attain stable deformed texture. Again, the as-FSP state shows similar range of SFs in all three alloys and deformed state shows similar range in all three alloys; but the spread in as-FSP state is different from that in tensile deformed state, because FSP induces partial deformation-induced grain rotation. Subsequently, tensile deformation involves further grain rotation. Therefore, the SFs characteristic of the as-FSP state correspond to the intermittent stage of grain rotation; and the set of SFs in the tensile deformed state corresponds to the stable end orientation of grains after further rotation.

Also, in all three alloys, SF of basal system is lower in as-FSP condition (<0.3) and increases from as-FSP to deformed condition (>0.3), while SF of prismatic slip decreases from 0.35–0.4 to <0.35. Pyramidal 〈a〉 and 〈c + a〉 slip systems have relatively higher SF (>0.4) in both as-FSP and deformed conditions. The observed SFs are attributed to the crystallographic texture along the tensile axis; specifically, in the as-FSP condition, several grains are oriented to favor prismatic and pyramidal slip (including 〈c + a〉 in the latter) rather than basal slip. However, as deformation proceeds, ε-fraction increases to >80%, and several ε-grains undergo rotation. Therefore, the deformed microstructure shows higher basal SF than as-FSP condition.

The relative SFs of the four slip modes for the three alloys in deformed condition are presented in Fig. 3(d). The average SFs for basal and prismatic slip are similar, while pyramidal 〈c + a〉 slip shows highest SF. Similar average SFs for basal and prismatic slip suggests that the stable end orientation (deformed texture) comprises equally distributed grains favorably oriented for basal and prismatic slip. This is also evident from SF distribution maps for basal slip (Fig. 3(e1–e3)) that show equally distributed grains with low SF and high SF. However, corresponding SF maps for pyramidal 〈c + a〉 slip (Fig. 3(e4–e6)) display uniformly distributed high SF, which is needed to accommodate strain according to von Mises criterion. In short, SF analysis suggests that FSP followed by tensile deformation induces a textural evolution (of the ε-phase) that is not entirely random but is rather a dual-textured microstructure where adjacent grains are preferentially oriented for either basal or prismatic slip; and higher strain is accommodated in both types of grains via pyramidal 〈c + a〉 slip.

**Figure 4:**
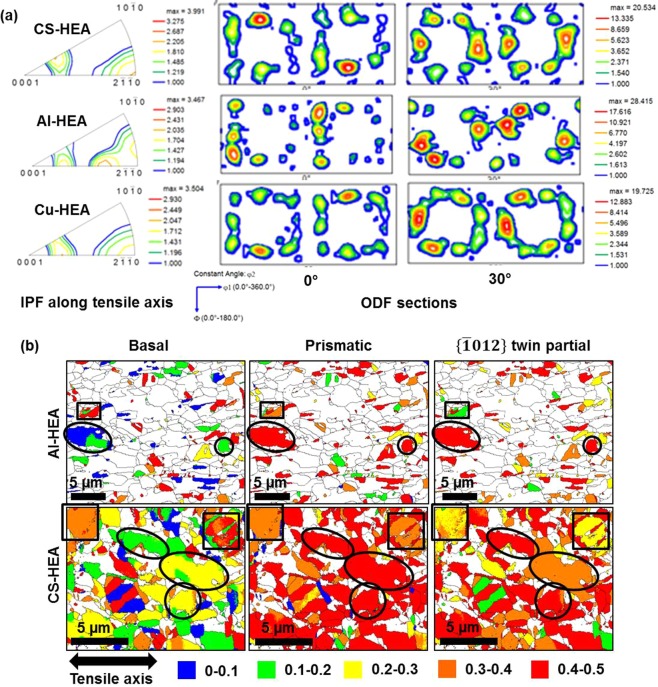
(**a**) Texture along the tensile axis for deformed specimens of CS-HEA, Al-HEA and Cu-HEA, represented as IPFs and ODF sections, and (**b**) schmid factors along tensile axis in as-FSP specimens of Al-HEA and CS-HEA to show the favorability of grains to undergo twinning.

The deformed texture evolution is confirmed from IPFs along the tensile axis and ODF sections (Fig. 4(a)). The IPFs clearly illustrate basal and away-from-basal orientations. The ODF sections (φ2 = 0° and 30° that are important for h.c.p. deformation texture^38,39^) also indicate the presence of two distinct texture components, thus confirming the dual-textured microstructure that accommodates initial strain by either basal or prismatic slip and higher strain by pyramidal 〈c + a〉 slip throughout the microstructure.

Deformation twinning is also important for accommodating strain along the c-axis^40,41^. Twin nucleation may take place by nonplanar dissociation of slip dislocations, initiating from either prismatic or basal 〈a〉 slip dislocations or partial basal dislocations^42^. Such dissociation would lead to the formation of a twinning partial and a stair rod (single dissociation) or leading and trailing twinning partials along with a stair rod dislocation (double dissociation). After the dissociation, the trailing partial dislocation remains on the glide plane and maintains an equilibrium distance with the stair rod. Also, the residual stair rod can successively produce more twinning dislocations, provided the leading partial glides sufficiently far away. The respective Burgers vectors of the leading twin partial for each twin system were obtained from the various dissociation reactions corresponding to all 7 possible h.c.p twin systems^42^, and these are summarized in Table 1.

**Table 1:** Burgers vectors of leading twin partial for various h.c.p. twin modes

Twin plane	Burgers vector	Miller indices	Remarks
$\{\bar{2}111\}$	〈a〉 + 2〈c〉	$\frac{1}{3}\langle \bar{2}116\rangle$	Single/double dissociation of PB or single dissociation of PP
$\{\bar{2}112\}$	〈a〉 + 〈c〉	$\frac{1}{3}\langle \bar{2}113\rangle$	Single/double dissociation of PB or single dissociation of PP
$\{\bar{2}113\}$	3〈a〉 + 2〈c〉	$\langle \bar{2}112\rangle$	Single/double dissociation of PB or single dissociation of PP
$\{\bar{2}114\}$	2〈a〉 + 〈c〉	$\frac{1}{3}\langle \bar{4}223\rangle$	Single/double dissociation of PB or single dissociation of PP
$\{\bar{1}011\}$	3〈d〉 + 2〈c〉	$\langle \bar{1}012\rangle$	Single/double dissociation of PB or pB, or single dissociation of PP
$\{\bar{1}012\}$	3〈d〉 + 〈c〉	$\langle \bar{1}011\rangle$	Single/double dissociation of PB or pB, or single dissociation of PP
$\{\bar{1}013\}$	9〈d〉 + 〈c〉	$\langle \bar{3}031\rangle$	Single/double dissociation of PB or pB
	9〈d〉 + 2〈c〉	$\langle \bar{3}032\rangle$	Single dissociation of PP

Note: 〈a〉 = $\frac{1}{3}\langle \bar{2}110\rangle$; 〈c〉 = $\langle 0001\rangle$; 〈d〉 = $\frac{1}{3}\langle \bar{1}010\rangle$; PB = Perfect Basal dislocation; pB = Partial Basal dislocation; PP = Perfect Prismatic dislocation.

Figure 4(b) presents an analysis of the favorability of grains (in the as-FSP microstructures of Al-HEA and CS-HEA) to undergo twinning by dissociation from basal or prismatic dislocations, based on resolved shear stress on the leading twin partial dislocation of $\{10\bar{1}2\}$ twinning, which is the most frequently observed twin mode in h.c.p. materials. Interestingly, grains (circled in black) showing lower Schmid factor for basal slip and higher SF for prismatic slip, exhibit higher SF for glide of the leading twin partial. In contrast, grains (outlined by black rectangles) showing higher SF for basal than prismatic slip, exhibit relatively lower SF for the twin partial glide. This confirms that leading twin partials resulting from prismatic dislocation dissociation are more favorably oriented for glide compared to leading twin partials emerging from basal dislocation dissociation. Therefore, in the present microstructures comprising alternate grains oriented favorably for basal and prismatic slip, the grains that are favorably oriented for prismatic slip are more likely to undergo subsequent twinning.

**Figure 5:**
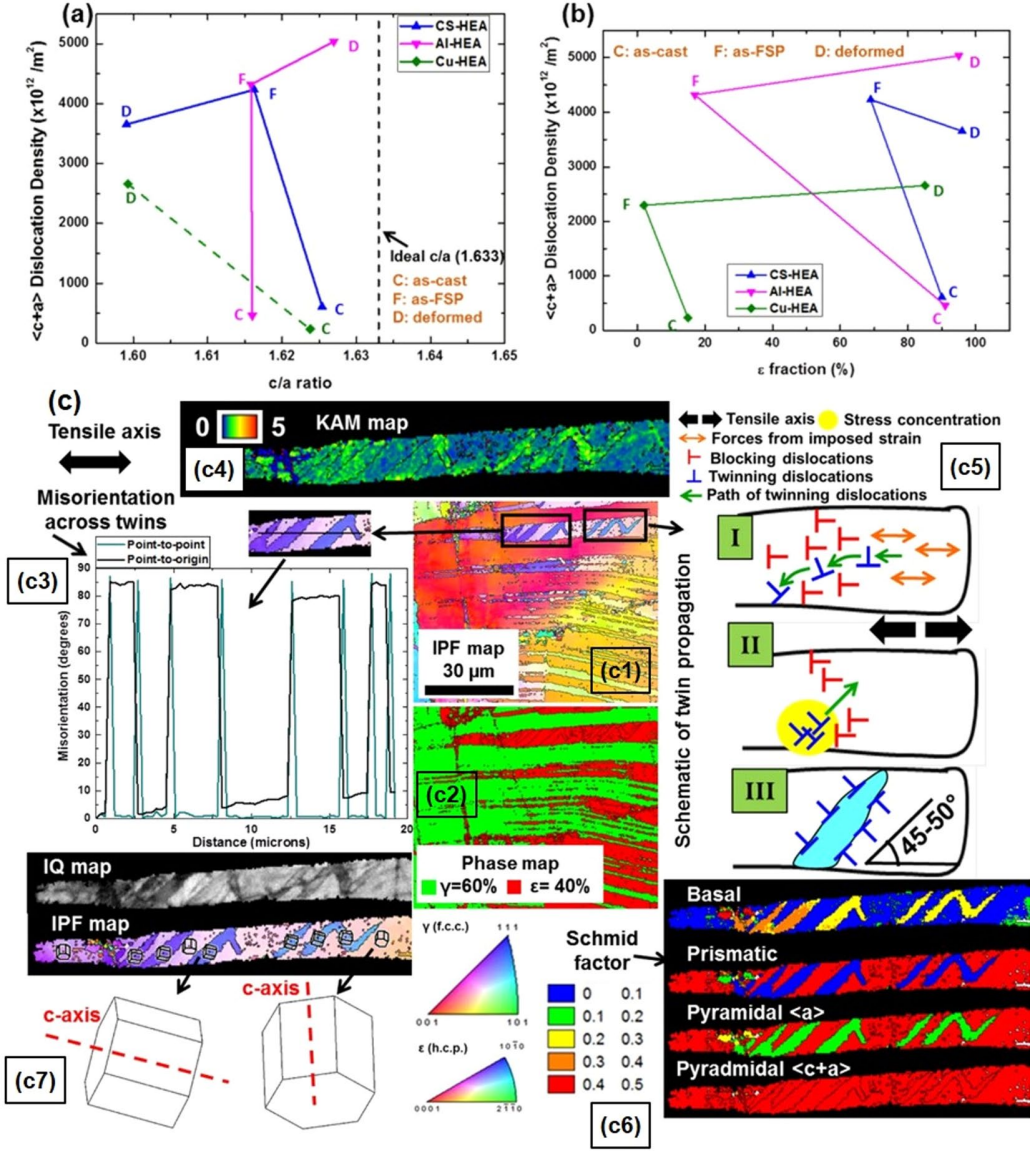
〈c + a〉 dislocation density as a function of (**a**) c/a ratio, and (**b**) ε fraction for CS-HEA, Al-HEA and Cu-HEA (Note: Cu-HEA is represented by dotted line in (**a**) because ε (h.c.p.) peak was not resolved in XRD of as-FSP specimen containing 2% ε); (**c**) deformation twinning in the ε (h.c.p.) phase in deformed specimen of as-cast Cu-HEA (c1) IPF map with twins outlined by black boxes, (c2) corresponding phase map, (c3) misorientation profile across twins, (c4) KAM map of ε-plate with twins, (c5) schematic to explain mechanism of twin-bridging, (c6) Schmid factor maps of ε-plate with twins, and (c7) IQ map and IPF map focusing on ε-plate with twins; unit cells denoting crystal orientation are marked on the IPF map with arrows pointing to enlarged image of unit cells.

Pyramidal 〈c + a〉 slip is important for work hardening or strain accommodation in ε (h.c.p.) phase. Hence, 〈c + a〉 dislocation densities (ρ_〈c+a〉_) were estimated. Figure 5(a,b) show ρ_〈c+a〉_ as functions of c/a ratio and ε-fraction, respectively, for CS-HEA, Al-HEA and Cu-HEA. Britton *et al*. discussed that the ease of basal slip decreases with decrease in c/a ratio in h.c.p. metals (except Be)^43^; d-electrons increase basal stacking fault energy (SFE) and promote prismatic dislocation dissociation that leads to prismatic slip. Yet, in Be, the absence of d-electrons decreases basal plane SFE and stabilizes dissociation into Shockley partials on the basal plane, thereby making basal slip easier despite low c/a ratio. Also, increasing Al content in Ti-Al alloys decreases the ratio of critical resolved shear stress (CRSS) of basal and prismatic slip and increases CRSS of 〈c + a〉 slip^43^. The latter argument suggests that 〈c + a〉 slip activity should be inhibited in Al-HEA on account of increased 〈c + a〉 CRSS. However, in the present study, both CS-HEA and Al-HEA show increase in ρ_〈c+a〉_ from as-cast to as-FSP condition with decrease in c/a. Also, Cu-HEA shows increased ρ_〈c+a〉_ with decreasing c/a from as-cast to deformed condition. These observations seem to be in line with the hypothesis that lower c/a hinders basal slip and favors 〈c + a〉 slip (while higher c/a hinders 〈c + a〉 slip). Surprisingly, Al-HEA and CS-HEA show counter-intuitive trends from as-FSP to tensile deformed condition. For Al-HEA, ρ_〈c+a〉_ increases with increasing c/a, while in CS-HEA, ρ_〈c+a〉_ decreases with decreasing c/a. This can be explained as follows. Note that, whatever the increase/decrease in c/a, the ε (h.c.p.) phase in CS-HEA and Al-HEA shows lower than ideal c/a ratio. Lower than ideal c/a inherently increases propensity for non-basal 〈c + a〉 slip and this is an important factor. Also, dual-textured microstructural evolution during FSP and tensile deformation induces equal distribution of grains favoring either basal or prismatic slip. When basal and prismatic slip saturate, 〈c + a〉 slip accommodates higher strain. In the intermittent deformed state, higher c/a ratio requires more strain accommodation along the c-axis. Therefore, the activity of 〈c + a〉 slip strongly depends on c/a ratio. Thus, during transition from the as-FSP to tensile deformed state, increasing c/a ratio in Al-HEA results in increased ρ_〈c+a〉_, while decreasing c/a in CS-HEA is manifest in decreased ρ_〈c+a〉_.

The ε-fraction dependence of ρ_〈c+a〉_ is much simpler to interpret (Fig. 5(b)). CS-HEA, Al-HEA and Cu-HEA show decrease in ε-fraction from as-cast to as-FSP state, but deformation induced by FSP causes increase in ρ_〈c+a〉_ in all three alloys. After tensile deformation, all three alloys transform to ε-dominant microstructures, wherein ρ_〈c+a〉_ increases similarly for Cu-HEA and Al-HEA; while ρ_〈c+a〉_ decreases slightly in CS-HEA due to decreased c/a ratio, as explained before. Overall, strain accommodation requirements overcome any phase fraction dependence of ρ_〈c+a〉_.

### Strain accommodation by the occurrence of deformation twinning

Our earlier studies confirmed that apart from 〈c + a〉 slip, deformation twinning is also important for strain accommodation^12,13^. In the present study, characteristic deformation twins were observed in the ε (h.c.p.) phase in tensile deformed specimens of as-cast Cu-HEA that illustrate the contribution of deformation twinning to strain accommodation. Figure 5(c) shows these twins on the inverse pole figure (IPF) map, phase map and image quality (IQ) map shown in Fig. 5(c1,c2,c7), respectively. The average twin thickness is 1.55 µm (±0.55). The misorientation angle corresponding to the twin boundaries is 86.2° (±1.2) (Fig. 5(c3)), which is similar to Mg (extension twin involves reorientation by an angle of 86.6°)^44^. Therefore, we can conclude that these are extension twins.

Figure 5(c4) shows that Kernel average misorientation (KAM) is higher inside the twins compared to outside the twins in the ε-plate, and confirms that twin formation is strongly influenced by the local stress state or strain distribution^37,45^. The crystallite orientations (denoted by unit cells marked on IPF map with arrow pointing to magnified image of the unit cell in Fig. 5(c7)) confirm that the martensite plate has c-axis oriented away from the tensile axis, while after reorientation due to twinning, the twinned regions have c-axis aligned close to the tensile axis.

The deformation twins show unique nucleation, propagation and growth characteristics that can be explained from the schematic in Fig. 5(c5). Twin nucleation depends strongly on local stress state. Due to the acicularity of the parent martensite plate, dislocations moving parallel to the tensile direction have greater mobility compared to dislocations attempting to move perpendicular to the length of the plate (or any other direction aligned at a large angle with the tensile direction). The movement of the latter dislocations is limited to approaching the phase boundaries on either side, where the available path is restricted by the heterogeneous phase interface. As a result, dislocations accumulate at periodic intervals along the martensite plate boundaries, and lead to local stress concentrations at these locations. Under the progressive application of strain, dislocations moving parallel to tensile direction stimulate stress fluctuations, and lead to nucleation of twins. Combined with the effect of crystal orientation, repeated nucleation of the same twin variant occurs. Local stress dependence of twin nucleation intuitively favors the formation of variously oriented twin fronts in polycrystalline microstructures comprising near equiaxed grains. However, in the present case, a double constriction effect acts on the available path for twinning dislocations. The sudden nearly perpendicular reorientation in the twinned volume would, in itself, orient the twin fronts of embryos so that they propagate perpendicular to the length of the martensite plate; however, free propagation in this direction is also restricted by the acicularity of the martensite plate, and the strain gradient along the tensile axis parallel to the length of the martensite plate introduces a translational effect on the movement of twinning dislocations at the twin interface. As a result, the only two possible twin front propagation directions are along 45–50° to the martensite boundary that is parallel to the tensile axis (clockwise and counterclockwise make two). Hence, this is the preferred orientation of the repeatedly nucleated twin variant, and results in twins inclined at 45–50° to the martensite plate. Thus, a most unusual ‘twin-bridging’ mechanism is observed. One twin propagates along 45–50° to reach the martensite boundary on the other side; then propagates 45–50° counterclockwise within the plate to return to the first boundary where it was nucleated; and, then, again propagates at 45–50° clockwise, with a resulting zig-zag twin across the breadth of the plate. While the c/a ratio and grain orientation dependence of twin boundary inclination (48.9° cutoff angle for Zr and 49.1° for Mg) were explained earlier by Arul Kumar *et al*.^46^ from simulations, the present work provides clear experimental evidence of the dependence of twin formation on the combination of geometrical criteria with material characteristics, CRSS, plastic deformation and c/a ratio.

The unique twin-bridging mechanism instead of lateral growth (twin thickening) is significant because twin-bridging represents the strain accommodation capability of the deformation twins. The driving force for twin growth is insufficient, yet twinning activity does not saturate because twinning is necessary to accommodate strain; the result is forced twin propagation in a preferred direction to accommodate strain across the width of the martensite plate. Further, Fig. 5(c6) shows that the Schmid factors of basal, prismatic and pyramidal 〈a〉 slip are low inside the twins (yellow, blue and green, respectively), while that of pyramidal 〈c + a〉 slip is high (red). Thus, after twin-bridging, the only mode of accommodating strain along c-axis within the twinned regions, is pyramidal 〈c + a〉 slip.

**Figure 6:**
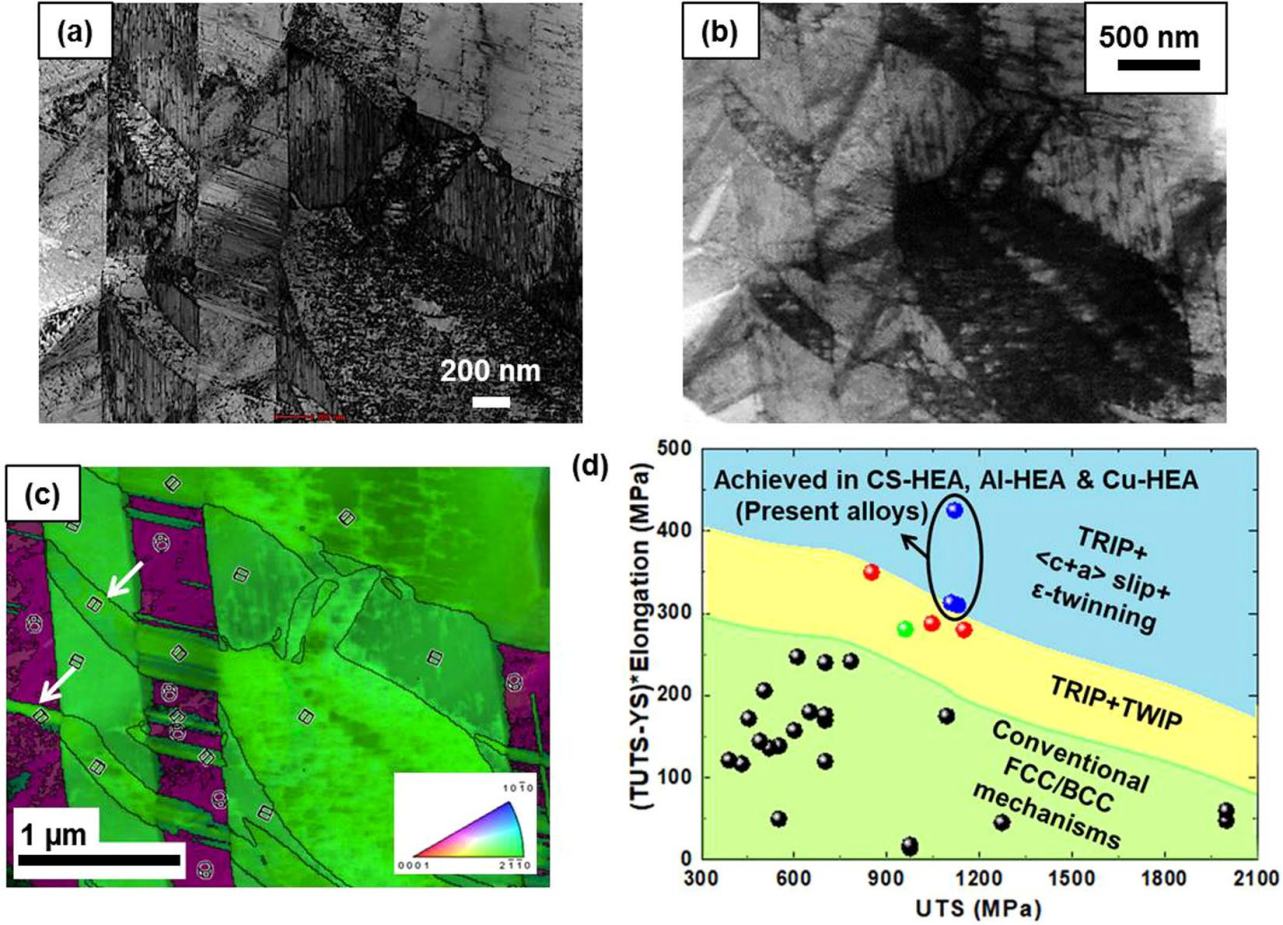
(**a**) TEM bright field image of nano-scale twins in as-cast CS-HEA, (**b**) corresponding virtual bright field image from PED. (**c**) OIM superimposed on virtual dark field image from PED to show different twin variants, and (**d**) schematic deformation mechanisms map to illustrate comparison of present HEAs with other HEAs in literature^8,10,12,13,47–60^.

While twin-bridging is a special case of strain accommodation by ε-twinning in tensile deformed specimen of as-cast Cu-HEA, the propensity for these TRIP-HEAs to form ε-twins is strongly connected to their alloy design induced microstructural constitution. For example, CS-HEA shows ε (h.c.p.) dominant microstructure in the as-cast condition itself. TEM characterization of as-cast CS-HEA revealed the presence of nano-scale twins in the microstructure (Fig. 6(a)). Further, precession electron diffraction (PED) confirmed the formation of primary and secondary twins of different variants (Fig. 6(b,c)). Note that, even twinning at the nano-scale exhibits interesting nucleation, propagation and growth tendency. One evidently observed example is the propagation of the twins marked by white arrows (Fig. 6(c)) through twin boundaries into the adjacent variant. The origin of such unique twin nucleation and propagation characteristics will be investigated in our future studies. Nevertheless, the present study unambiguously confirms the importance of ε-twinning in our TRIP-HEAs and also reveals that ε-twins nucleated in these alloys often undergo unconventional propagation/growth. The latter propensity increases the probability of achieving high work hardening through ε-twinning.

### Overview of deformation mechanisms in HEAs

The present study showed that transformative HEAs use multiple deformation mechanisms to accommodate strain, justifying their excellent mechanical response discussed in previous studies^12,13^. A schematic deformation mechanisms map comparing these HEAs with various HEAs in literature illustrates the various strength-ductility regimes (Fig. 6(d)).

The strength-ductility index (SDI = (true UTS - YS) × uniform elongation) is plotted against UTS to summarize SDI-UTS combinations achieved for various HEAs. Earlier work by Li *et al*.^8,47^ confirmed that using multiple mechanisms like TRIP and TWIP is a pathway to overcome the strength-ductility trade-off. We achieved the objective of increased SDI by activating multiple deformation mechanisms by combining metastability-based alloy design with thermomechanical processing (FSP route) induced microstructural flexibility. Exceptional SDI-UTS was possible in our CS-HEA, Al-HEA and Cu-HEA alloys by combining conventional mechanisms like grain refinement with TRIP effect and h.c.p. deformation mechanisms.

## Summary

The present study showed that the ε (h.c.p.) phase in transformative HEAs exhibits c/a ratio similar to Mg in the as-cast condition. The c/a ratio and transformation volume are responsive to alloy chemistry, microstructure and stress state. Microstructural evolution of the ε-phase during FSP followed by deformation is such that a dual texture is developed with progressive strain, wherein alternate grains orient themselves for basal and prismatic slip to accommodate initial strain; subsequently, higher strain is accommodated by pyramidal 〈c + a〉 slip and deformation twinning. Therefore, these alloys use multiple deformation mechanisms to achieve excellent strength-ductility index. Also, a unique twin-bridging phenomenon was observed that experimentally proved earlier theoretical predictions of characteristic twin boundary inclination.

## Methods

The five HEAs studied in the present investigation, their alloy designations and nominal compositions are shown in Table 2. The theoretical background for composition design (of these alloys) based on phase diagrams was discussed in detail in a previous study by Nene *et al*.^10^. The alloys were produced by vacuum arc-casting in a cold copper crucible, using pure metals and ingot dimensions of 300 × 100 × 6 mm^3^. The chamber was backfilled with argon to 1 atm. prior to each melt.

**Table 2:** Nominal compositions of HEAs in the present study.

Alloy Designation	Nominal composition (at. %)
Si3-HEA	Fe_42_Mn_30_Co_10_Cr_15_Si_3_
Si5-HEA	Fe_42_Mn_28_Co_10_Cr_15_Si_5_
CS-HEA	Fe_40_Mn_20_Co_20_Cr_15_Si_5_
Al-HEA	Fe_39_Mn_20_Co_20_Cr_15_Si_5_Al_1_
Cu-HEA	Fe_38.5_Mn_20_Co_20_Cr_15_Si_5_Cu_1.5_

Friction stir processing (FSP) was performed on the as-cast HEA sheets using a tungsten-rhenium (W-Re) processing tool with 12 mm shoulder diameter with tapered pin, 7.5 mm root diameter, 6 mm pin tip diameter and 3.5 mm pin length. Double-pass (D-pass) FSP was performed with tool rotation rate of 350 rotations/min (rpm) in the first pass, followed by a second overlapping pass at 150 rpm. The traverse speed and tilt angle were 50.8 mm/min and 2°, respectively. The plunge depth in the first and second pass was 3.65 mm and 3.70 mm, respectively.

Flat (rectangular) dogbone-shaped mini-tensile specimens with gage length 5 mm, width 1.25 mm and thickness 1 mm were machined out using a mini computer numerical control machine from 1 mm below the surface from the stirred region of the as-FSP material. Room-temperature tensile tests to failure were carried out in a mini-tensile tester at initial strain rate of 10^−3^ s^−1^.

X-ray diffraction (XRD) measurements of as-cast, as-FSP and tensile-deformed specimens were performed with a Rigaku Ultima III diffractometer and Cu Kα radiation operating at 40 kV and 44 mA. Lattice parameters of γ (f.c.c.) and ε (h.c.p.) phases and c/a ratio of ε (h.c.p.) phase were calculated from XRD peak positions using the following procedure. The peak positions (2θ) were noted and interplanar spacing d_hkl_ was calculated from Bragg’s law using,

5 $${{\rm{d}}}_{{\rm{hkl}}}=\frac{{\rm{\lambda }}}{2\,\sin \,{\rm{\theta }}}$$

where λ is the wavelength of X-ray. The relationship between d_hkl_ and lattice parameters for γ (f.c.c.) and ε (h.c.p.) crystal structures are given by Eqs (6) and (7), respectively.

6 $$\frac{1}{{({{\rm{d}}}_{{\rm{hkl}}})}_{{\rm{fcc}}}}=\frac{\sqrt{{{\rm{h}}}^{2}+{{\rm{k}}}^{2}+{{\rm{l}}}^{2}}}{{{\rm{a}}}_{{\rm{fcc}}}}$$

7 $$\frac{1}{{({{\rm{d}}}_{{\rm{hkl}}})}_{{\rm{hcp}}}^{2}}=\frac{4}{3}(\frac{{{\rm{h}}}^{2}+{\rm{hk}}+{{\rm{k}}}^{2}}{{{\rm{a}}}_{{\rm{hcp}}}^{2}})+\frac{{{\rm{l}}}^{2}}{{{\rm{c}}}_{{\rm{hcp}}}^{2}}$$

The (111) and (200) peaks were used to obtain a_fcc_ for the γ (f.c.c.) phase and (002) and (100) peaks were used to obtain c_hcp_ and a_hcp_, respectively, of the ε (h.c.p.) phase. The lattice parameters c_hcp_ and a_hcp_ so obtained were used to calculate c/a ratio of the ε (h.c.p.) phase.

Transmission electron microscopy (TEM) specimens of CS-HEA in as-cast and D-pass FSP conditions were prepared by focused ion beam (FIB) milling process using FEI Nova 200 NanoLab Dual Beam FIB/FESEM. TEM characterization was carried out using a FEI Tecnai G2 F20 S-Twin 200 keV field emission TEM. The c/a ratio of ε (h.c.p.) phase in as-cast CS-HEA was also verified from TEM diffraction pattern (DP). The procedure for obtaining c/a ratio from TEM DP with $[11\bar{2}0]$ zone axis is as follows,

8 $$\frac{1}{{{\rm{d}}}_{{\rm{hkl}}}^{2}}=\frac{{{\rm{R}}}_{{\rm{hkl}}}^{2}}{{{\rm{\lambda }}}^{2}{{\rm{L}}}^{2}}$$

where R_hkl_ is the distance of the diffraction spot corresponding to (hkl) from the central spot, λ is the wavelength of electron and L is the camera length of the TEM. Using Eq. (7), we get the relationships,

9 $$\frac{1}{{{\rm{d}}}_{001}^{2}}=\frac{1}{{{\rm{c}}}_{{\rm{hcp}}}^{2}}$$

10 $$\frac{1}{{{\rm{d}}}_{100}^{2}}=\frac{4}{3{{\rm{a}}}_{{\rm{hcp}}}^{2}}$$

Now, using Eqs (8–10), we can get c/a ratio,

11 $$\frac{{{\rm{c}}}_{{\rm{hcp}}}}{{{\rm{a}}}_{{\rm{hcp}}}}=\frac{\sqrt{3}}{2}\frac{{{\rm{R}}}_{100}}{{{\rm{R}}}_{001}}$$

TEM based OIM - PED was carried out on as-cast CS-HEA TEM foil, to observe nano-scale ε (h.c.p.) twins, prepared using FEI Nova 200 NanoLab Dual Beam FIB/FESEM. The data was acquired on FEI Tecnai G2 F20 S-Twin 200 keV using NanoMEGAS system. The parameters were set at C2 aperture of 30 µm, spot size of around 2 nm (spot size of 8 on the FEI system), camera length of 135 mm and step size of 10 nm. The data acquired using TOPSPIN 3.0 software was analyzed by ACOM software where reference bank of diffraction patterns were compared with the current data, resulting in OIM information presented in Fig. 6(c).

Microstructural characterization by electron backscatter diffraction (EBSD) of as-cast, as-FSP and tensile-deformed specimens was performed using a FEI Nova Nano SEM 230 equipped with Hikari Super EBSD detector at an operating voltage of 20 kV. TEAM^TM^ software enabled data acquisition and TSL OIM Version 8 software was used for data analysis. Typical step sizes of EBSD acquisition at various magnifications were 0.9 µm, 0.2 µm and 0.08 µm at 500 × (300 µm × 238 µm), 2000 × (75 µm × 60 µm) and 6000 × (25 µm × 20 µm), respectively. Phase fractions reported from EBSD were obtained by averaging three or more scans.

## Supplementary information

On the evolving nature of c/a ratio in a hexagonal close-packed epsilon martensite phase in transformative high entropy alloys

## Data Availability

The raw/processed data required to reproduce these findings cannot be shared at this time, as the data are also part of other ongoing studies.
