# Supplementary material for: On the evolving nature of c/a ratio in a hexagonal close-packed epsilon martensite phase in transformative high entropy alloys
Source: Sci Rep. 2019 Sep 12;9:13185. doi: 10.1038/s41598-019-49904-5 (PMC6742669; doi:10.1038/s41598-019-49904-5)
Supplement: Supplementary file 1 — On the evolving nature of c/a ratio in a hexagonal close-packed epsilon martensite phase in transformative high entropy alloys [file 41598_2019_49904_MOESM1_ESM.docx]

**Supplementary Information**

**On the evolving nature of c/a ratio in a hexagonal close-packed epsilon martensite phase in transformative high entropy alloys**

Subhasis Sinha^1^, Saurabh S. Nene^1^, Michael Frank^1^, Kaimiao Liu^1^, Priyanka Agrawal^1^, Rajiv S. Mishra^1*^

**Table S1: Complete list of measured lattice constants and phase fractions for the various alloys in their different conditions in the present study**

| **Alloy** | **Condition** | **Phase fraction** | | **c_hcp_** | **a_hcp_** | **(c/a)_hcp_** | **a_fcc_** |
| --- | --- | --- | --- | --- | --- | --- | --- |
| Si3-HEA | As-cast | 98% γ | 2% ε | 4.1313 | 2.5411 | 1.6258 | 3.6077 |
|  | As-FSP | 95% γ | 5% ε | 4.1389 | 2.5288 | 1.6367 | 3.6075 |
|  | Deformed | 90% γ | 10% ε | 4.1251 | 2.5272 | 1.6323 | 3.6094 |
| Si5-HEA | As-cast | 91% γ | 9% ε | 4.1326 | 2.548 | 1.6219 | 3.6112 |
|  | As-FSP | 91% γ | 9% ε | 4.0949 | 2.5444 | 1.6094 | 3.6066 |
|  | Deformed | 90% γ | 10% ε | 4.1449 | 2.5216 | 1.6438 | 3.608 |
| CS-HEA | As-cast | 10% γ | 90% ε | 4.1272 | 2.5392 | 1.6254 | 3.5916 |
|  | As-FSP | 31% γ | 69% ε | 4.1151 | 2.5461 | 1.6162 | 3.5885 |
|  | Deformed | 4% γ | 96% ε | 4.0712 | 2.546 | 1.5991 | 3.5836 |
| Al-HEA | As-cast | 9% γ | 91% ε | 4.1046 | 2.54 | 1.616 | 3.5811 |
|  | As-FSP | 83% γ | 17% ε | 4.1155 | 2.547 | 1.6158 | 3.606 |
|  | Deformed | 5% γ | 95% ε | 4.1218 | 2.5334 | 1.627 | 3.5894 |
| Cu-HEA | As-cast | 85% γ | 15% ε | 4.1257 | 2.5408 | 1.6238 | 3.5969 |
|  | As-FSP | 98% γ | 2% ε | NA* | | | 3.5957 |
|  | Deformed | 15% γ | 85% ε | 4.0819 | 2.5524 | 1.5992 | 3.5919 |

*epsilon peaks were not obtained in XRD of this condition with 2% epsilon fraction
